# Tibiofemoral and patellofemoral joint 3D-kinematics in patients with posterior cruciate ligament deficiency compared to healthy volunteers

**DOI:** 10.1186/1471-2474-13-231

**Published:** 2012-11-26

**Authors:** Ruediger von Eisenhart-Rothe, Ulrich Lenze, Stefan Hinterwimmer, Florian Pohlig, Heiko Graichen, Thomas Stein, Frederic Welsch, Rainer Burgkart

**Affiliations:** 1 Department of Orthopaedics and Orthopaedic Sports Medicine, Technische Universität München, Klinikum Rechts der Isar; Ismaninger Str. 22, Munich D-81675, Germany; 2 Asklepios Orthpädische Klinik Lindenlohe, Lindenlohe 18, Schwandorf, 92421, Germany; 3 Berufsgenossenschaftliche Unfallklinik Frankfurt am Main, Friedberger Landstrasse 430, Frankfurt am Main, 60389, Germany

**Keywords:** Kinetics, PCL deficiency, Magnetic resonance imaging, Femorotibial translation, Three dimensional, 3D

## Abstract

**Background:**

The posterior cruciate ligament (PCL) plays an important role in maintaining physiological kinematics and function of the knee joint. To date mainly in-vitro models or combined magnetic resonance and fluoroscopic systems have been used for quantifying the importance of the PCL. We hypothesized, that both tibiofemoral and patellofemoral kinematic patterns are changed in PCL-deficient knees, which is increased by isometric muscle flexion. Therefore the aim of this study was to simultaneously investigate tibiofemoral and patellofemoral 3D kinematics in patients suffering from PCL deficiency during different knee flexion angles and under neuromuscular activation.

**Methods:**

We enrolled 12 patients with isolated PCL-insufficiency as well as 20 healthy volunteers. Sagittal MR-images of the knee joint were acquired in different positions of the knee joint (0°, 30°, 90° flexion, with and without flexing isometric muscle activity) on a 0.2 Tesla open MR-scanner. After segmentation of the patella, femur and tibia local coordinate systems were established to define the spatial position of these structures in relation to each other.

**Results:**

At full extension and 30° flexion no significant difference was observed in PCL-deficient knee joints neither for tibiofemoral nor for patellofemoral kinematics. At 90° flexion the femur of PCL-deficient patients was positioned significantly more anteriorly in relation to the tibia and both, the patellar tilt and the patellar shift to the lateral side, significantly increased compared to healthy knee joints. While no significant effect of isometric flexing muscle activity was observed in healthy individuals, in PCL-deficient knee joints an increased paradoxical anterior translation of the femur was observed at 90° flexion compared to the status of muscle relaxation.

**Conclusions:**

Significant changes in tibiofemoral and patellofemoral joint kinematics occur in patients with isolated PCL-insufficiency above 30 degrees of flexion compared to healthy volunteers. Since this could be one reasonable mechanism in the development of osteoarthritis (OA) our results might help to understand the long-term development of tibiofemoral and/or patellofemoral OA in PCL-insufficient knee joints.

## Background

The posterior cruciate ligament (PCL) plays an important role in maintaining physiological kinematics and function of the knee joint [1-3]. Long term results suggest that PCL-insufficiency might promote the development of knee joint osteoarthritis (OA) due to an excessive displacement between tibia and femur [4-6]. In this context up to 80% of patients with a symptomatic PCL insufficiency of more than 5 years show significant cartilage damage in the medial knee compartment and up to 50% in the femoro-patellar joint [7].

To date mainly in-vitro models [8-12] or combined in-vivo magnetic resonance and fluoroscopic systems [13,14] have been used for quantifying the importance of the PCL as a passive joint stabilizer and the analysis of tibiofemoral translation and rotation patterns in humans. However, in-vitro models bare the handicap of limited muscle simulation resulting in an impairment of their significance with regard to the in-vivo situation. Fluoroscopic systems in contrast suffer from radiation exposure to the patients. In this study patients were examined in an open MR system under flexing isometric muscle activity and 3D kinematics of the tibiofemoral and patellofemoral joints were analyzed by 3D image postprocessing.

Although the influence of tibiofemoral kinematics on patellar motion especially in patients with instability of the knee has been impressively demonstrated, only few studies exist analyzing the patellofemoral kinematics in PCL-deficient patients [10,14-17]. However, in vivo studies on tibiofemoral kinematics in patients with PCL-insufficiency reported on an increased posterior and lateral translation as well as a decreased internal rotation of the tibia relative to the femur beyond 30° of knee flexion [13,18].

Since this could be a reasonable mechanism in the development of OA in patients with PCL deficiency we aimed in this study the simultaneous investigation of tibiofemoral and patellofemoral kinematics of PCL deficient knee joints in comparison to healthy volunteers.

Main hypothesis of this study was, that in PCL deficient knee joints:

a) Tibiofemoral motion patterns are significantly changed in terms of an increased posterior translation and external rotation of the tibia at knee flexion above 30°.

b) Patellofemoral kinematics are changed showing an increase of the patellar shift and tilt angle

c) Isometric muscle flexion due to an extending torque leads to an increase of a) and b).

## Methods

### Subjects

In this prospective cohort study we enrolled 12 patients (8 males, 4 females; age 20–34 years) with isolated insufficiency of the PCL prior to anterolateral single bundle PCL augmentation as well as a control group of 20 healthy volunteers (14 males; 6 females; age 18–36 years) without any history of knee-pain, -trauma or -surgery.

All patients had, 7–12 months after the initial trauma and either failed diagnosis or failed conservative treatment, clinical and radiological symptoms of posterior but not postero-lateral instability (posterior tibial sag sign; positive quadriceps active test and posterior drawer test; positive PLRI - dial test at 90° of knee flexion and negative at 30°; normal varus/valgus stress tests; all clinical test were assessed by the corresponding author). None of the patients had radiological signs of OA or suffered from load-dependent knee pain. A high field MR-examination performed prior to the study showed no degenerative cartilage lesions or additional traumatic alterations (e.g. bone bruise or cartilage damage).

The ethics committee of the Goethe University Frankfurt approved all parts of the study. Written informed consent was obtained from all patients and volunteers enrolled prior to MR imaging.

### MR imaging acquisition

**Figure 1:**
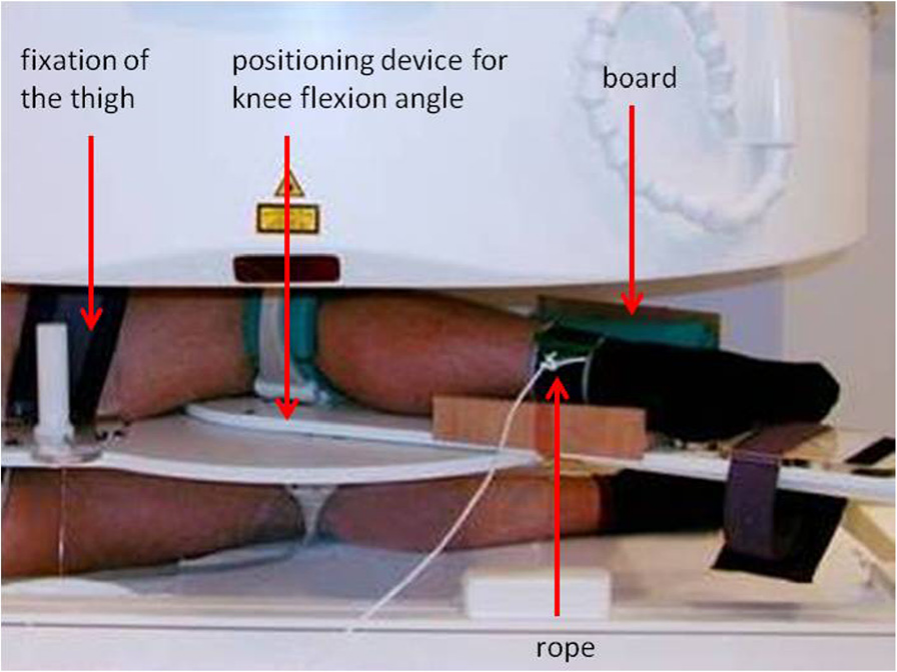
**Photograph showing an examination setup using an open MR: The patient is placed in lateral position with the examined knee on the top.** The knee flexion angle is controlled with a special *positioning device* which does not interfere with MR-scans. To ensure that the knee remains in a constant position the upper third of the *thigh is fixed* to the device. Additionally, during image acquisition the shank needs to be in contact with a *board* installed onto the position device. The torque can be applied using a *rope* and a pulley. (consider: in the shown setup the torque will be applied in flexing direction!).

Kinematics analysis was performed using an open MR system (0.2 T; Magnetom-Open, Siemens Medical Solutions, Erlangen, Germany) as previously published [17,19]. Images were acquired by means of a 3D GRE sequence (LR-flash: TR 18; TE 5ms; FA 30°; FoV 220^2^; matrix 128x256) with an acquisition time of 4 minutes and 26 seconds. Subjects were placed in lateral position with the injured knee on the top. The knee joints were positioned in three different flexion angles (0°, 30°, 90°). The knee flexion angle was controlled with a special positioning device (Siemens Medical Solutions, Erlangen, Germany), which did not interfere with MR-scans and allowed a reproducible adjustment of the knee at different flexion angles under avoidance of motion artefacts (Figure 1). To ensure that the knee remained in a constant position the upper third of the thigh was fixed to the positioning device. Additionally a board was installed onto the positioning device to which the shank needed to be in contact during image acquisition (Figure 1). The positioning device did neither affect tibial rotation nor translation. In all three positions a weight of 3 kg was applied to the lower third of the shank (35 cm distal to the knee joint space), to produce a torque of 10 Nm. This torque was applied in an extending direction. Thus, isometric muscle activity of the flexor muscles was produced. The rationale was to increase potentially pathologic kinematics in PCL deficient knee joints. The torque was applied in the plane of the leg perpendicular to the axis of the tibia using a nylon rope and a pulley. Isometric muscle activity over the entire acquisition period was verified by surface electromyography of the flexor muscle groups prior to MR imaging. Thus, continuous isolated activity of the muscles was assured according to the direction of the torque.

### Image analysis

All MR imaging data were transferred onto a parallel computing system (Octane Duo, Silicon Graphics, and Mountain View, CA). For kinematics analysis, semi-automated segmentation of the femur, tibia and patella was performed [17,19,20]. After a tri-linear interpolation, all anatomic structures were reconstructed in three dimensions.

**Figure 2:**
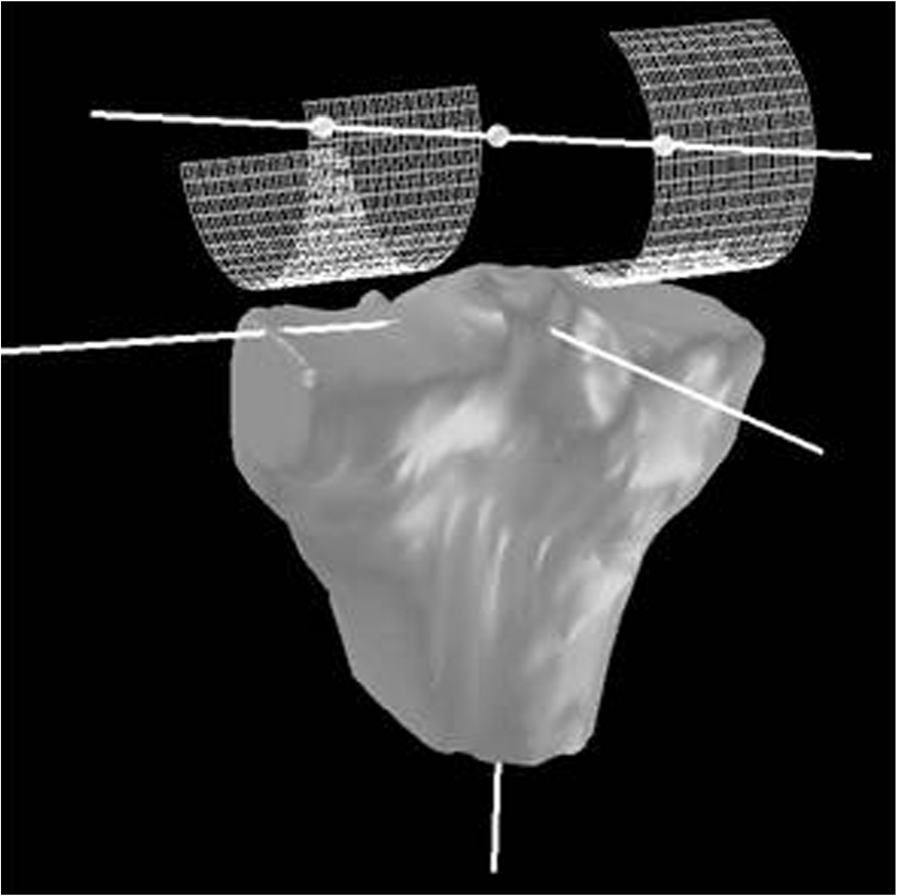
The epicondylar axis, which was determined using a cylinder fitting technique with reference points for the medial and lateral femoral condyle and its femoral centre, was projected into the tibia-based coordinate system.

A tibia-based local coordinate system was calculated to determine the position of the patella and the femoral condyles in relation to the tibia as well as the amount and direction of the displacement between the different image acquisitions [17,19]. Therefore the articular surface of the tibial plateau was interactively segmented on each slice, and the centre of mass (area-centroid) of the tibial plateau was computed. Based on its spatial orientation, a 3D local coordinate system was determined, with its origin in the centre of mass (area-centroid) of the tibial plateau (Figure 2).

To quantify tibiofemoral kinematics, femoral reference points were defined which remained unaffected during knee flexion. Therefore a cylinder fitting technique was applied to determine the epicondylar axis (Figure 2) with reference points for the medial and lateral femoral condyle and its femoral centre, respectively [17,19]. Finally this epicondylar axis was projected onto the tibial coordinate system to calculate both, femoral translation and rotation, between the different image acquisitions (Figure 2).

**Figure 3:**
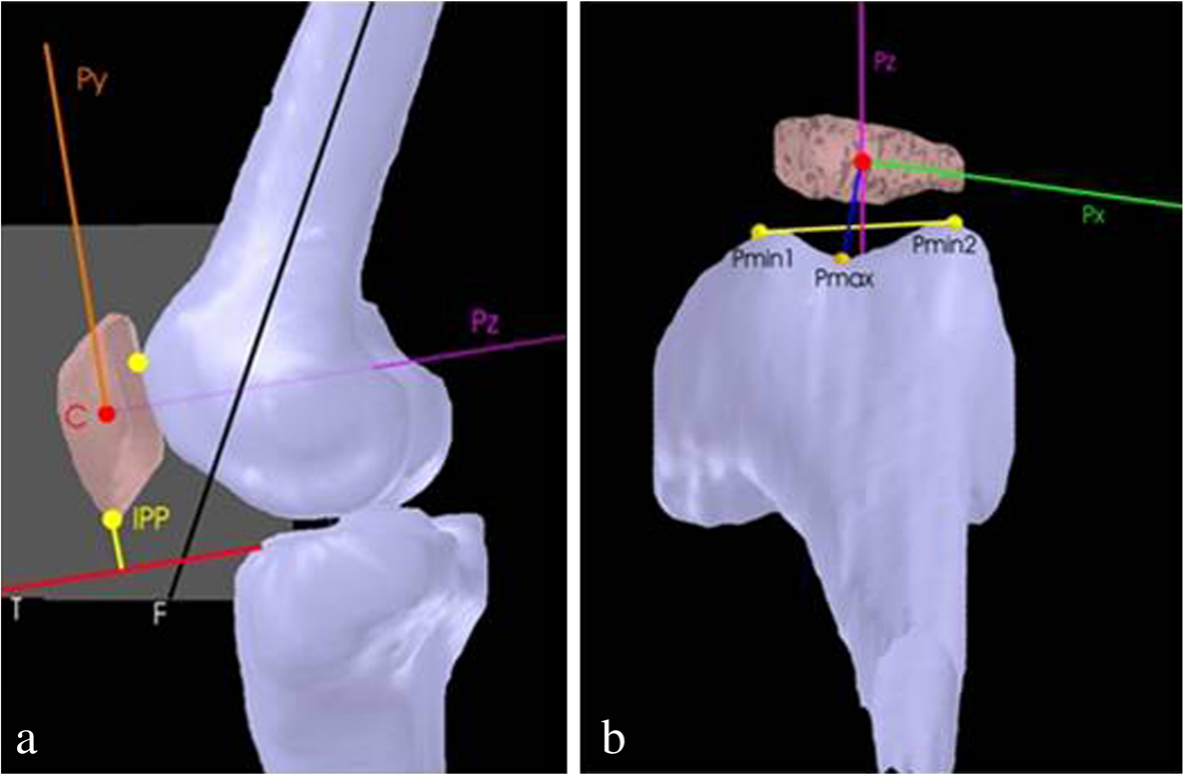
**Patella-based coordinate system.a**) (Sagittal view): C: Center of Patella; Py: y-axis of patella; T: x-axis of tibia plateau; F: longitudinal axis of the femur; IPP inferior patella pole. Patellar height: distance between IPP and T. Patello-femoral angle: Angle between Py and F. **b**) (Transversal view) Pmin1 and Pmin2: Shortest distances of the sulcus to the y-z-plane (x=0); Pmax: Largest distance of the patella sulcus to the y-z-plane (x=0). Tilt angle: Angle between line Pmin1 - Pmin2 and Px (x-axis of patella). Patellar shift: Displacement between C and Pmax, projected on T.

For the analysis of the patellofemoral kinematics a patella-based local coordinate system (PBCS) with the origin of the PBCS in the computed centre of the patella was calculated, based on the interpolated segmented voxel data (Figure 3). Femoral reference points were defined in three dimensions in order to quantify the orientation and position of the patella in relation to the femoral trochlear groove (Figure 3). Finally, the position of these femoral reference points was projected onto the PBCS allowing a 3D determination out of the established 2D parameters for describing patellofemoral kinematics [21,22]. The high reproducibility and validity of this method was tested and described in a previous experiment [17,19].

### Statistical analysis

The effect of three investigated factors (F1: PCL-deficiency; F2: Knee flexion; F3: flexor muscle activity due to application of an extending torque) on the position of the reference points relative to the tibia were assessed by mixed models regression analysis using an exchangeable correlation matrix to take the occurrence of repeated measures into account. All tests were two-sided and conducted in an explorative manner using SPSS (Statistical Package for the Social Sciences, version 19.0).

The influence of the joint position was determined by comparing the values of one flexion position with the following, each with and without flexing muscle activity. The influence of muscle activity was determined by comparing the values with and without muscle activity in one specific joint position. First the position of the femur relative to the tibia was calculated for each group and the effect of the two investigated factors knee position and muscle activity were determined using the student’s t-test for paired groups. Thereafter the data from the patients group was compared with the data from the healthy subjects using the student’s t-test for unpaired groups (Statview 4.5, Abacus Concepts, Berkeley, CA). A p-value of <0.05 was considered as statistical significant.

## Results

Effect of PCL-deficiency (F1), knee flexion (F2) and flexor muscle activity (F3) on the position of the medial and lateral femoral condylus, the central reference point and the epicondylar axis.

Isometric contraction of the flexor muscle group (p=0,003) as well as a flexed knee position (p=0,036) had a significant effect on the position of the medial femoral condyle. PCL-deficiency (p<0,001) as well as knee flexion (p<0,001) significantly influenced the position of the lateral condyle reference point, whereas isometric contraction of the flexor muscle group of the knee did not (p=0,06). The position of the central reference point was significantly changed in PCL-deficient knees (p=0,001) and during knee flexion (p<0,001). Rotation of the epicondylar axis was significantly influenced by isometric contraction of the flexor muscle group (p<0,001) as well as knee flexion (p<0,001). PCL-deficiency did not have a statistically significant effect on the rotation of the epicondylar axis.

### Tibiofemoral kinematics

**Figure 4:**
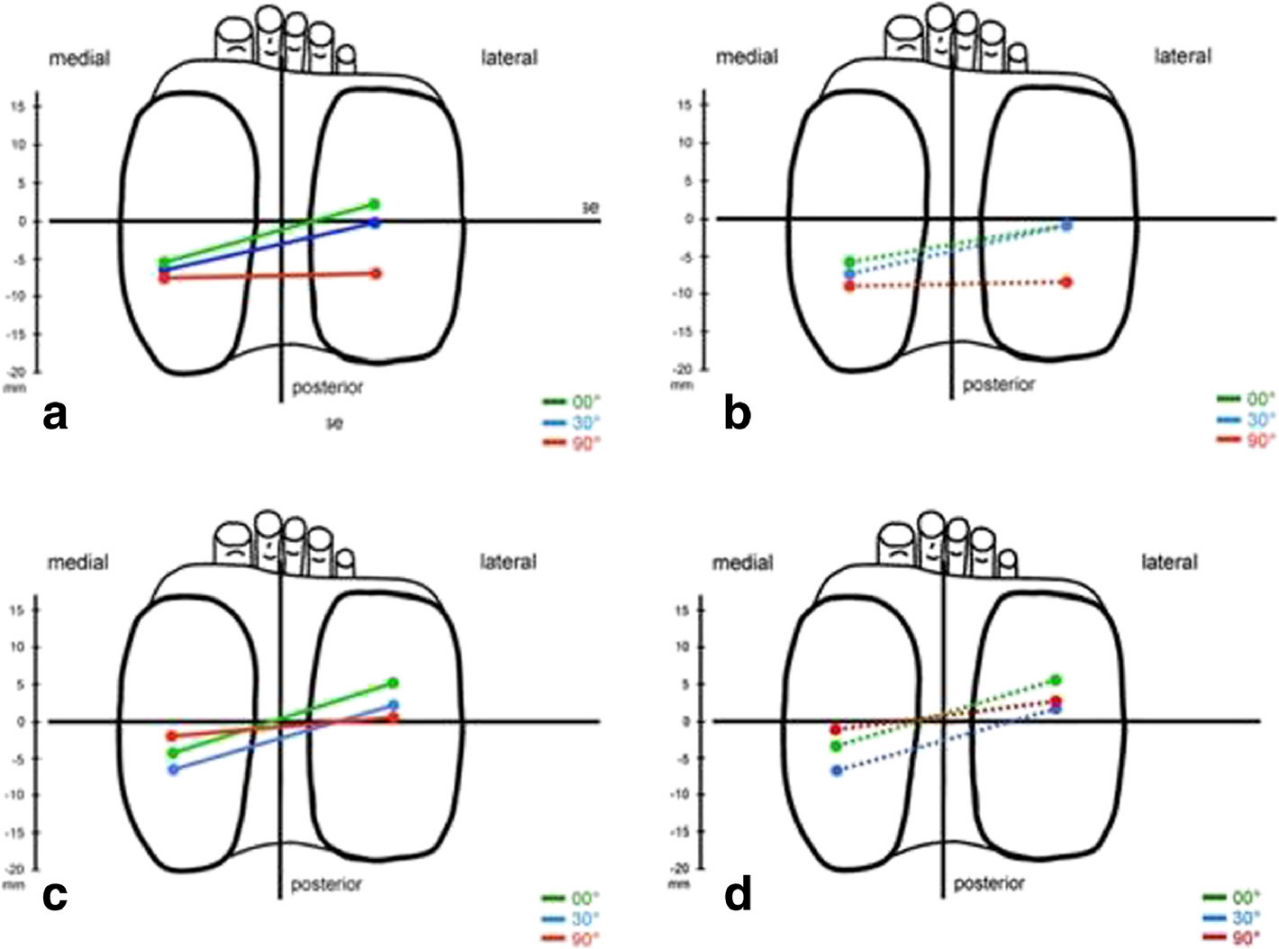
Tibiofemoral translation and rotation at 0°, 30° and 90° knee flexion in healthy knees (a) without and (b) with flexing muscle activity, and in PCL-deficient knees (c) without and (d) with flexing muscle activity (femoral line represents the transepicondylar axis).

The movement of all healthy knee joints started from a characteristic internally rotated position of the femur with respect to the tibia in full extension. From this position a typical femoral rollback during knee joint flexion was observed (Figure 4a). Due to the additional external rotation of the transepicondylar axis from 30° to 90° flexion, the amount of translation reached higher values for the lateral condyle. This resulted in a significantly (p<0.05) more posterior position of the central and lateral femoral reference points in relation to the tibia at 30° and 90° flexion compared to full extension. No significant changes for translation and rotation were observed comparing the status with and without isometric muscle activity (Figure 4b).

PCL-deficient knee joints showed no significant changes compared to healthy knee joints at full extension and 30° flexion. The starting position was an internally rotated femur in extension with a posterior translation from 0° to 30° flexion in PCL deficient and healthy knees, respectively. At 90° flexion the femur was more anteriorly positioned compared to the knee joint of healthy volunteers (*P* < 0.05). Due to the additional external rotation from 30° to 90° flexion the position of the lateral femoral condyle remained almost constant while the medial condyle demonstrated a paradoxical anterior translation of more than 5mm (Figure 4c). During the whole range of motion the amount of translation was increased medially and significantly reduced laterally, leading to a decreased femoral external rotation during knee flexion. In PCL deficient knee joints flexing muscle activity had no significant influence on translation and rotation at full extension and 30° flexion. At 90° flexion the amount of paradoxical anterior translation was higher compared to muscular relaxation leading to a more anteriorly positioned femur in relation to the tibia at 90° flexion (Figure 4d).

### Patellofemoral kinematics

In the sagittal plane during flexion the healthy knee joints presented a significant increase of patellar height and patellofemoral angle (p < 0.05) (Table 1). In the transversal plane the patellar tilt (angle open to the medial side) and the lateral patellar shift remained almost constant from 0 to 30° flexion. From 30 to 90° flexion, the patellar tilt decreased significantly whereas the lateral patellar shift increased significantly (p < 0.05; Table 1). During activity of the flexor muscle group the patellar height and the patellar shift (to the lateral side) increased in all investigated knee joint positions compared to the results during muscle relaxation, but these results did not reach statistical significance.

**Table 1:** Patellofemoral kinematics in healthy knees and knees with PCL-insufficiency (mean values ± standard deviation) during knee flexion (0-90°)

	**0°**	**0°**	**30°**	**30°**	**90°**	**90°**
		**flex. muscles**		**flex. muscles**		**flex. muscles**
Patellar height [mm]	16.2 ± 3.8	19.7 ± 4.9	17.0± 6.3	17.2 ± 3.6	23.5 ± 3.2	23.7 ± 7.6
Femoro-patellar angle [°]	5.9 ± 5.2	7.8 ± 4.6	21.2 ± 8.2	16.3 ± 10.8	49.9 ± 6.3	52.2 ± 7.8
Tilt angle[°]	8.7 ± 3.4	9.7 ± 4.1	9.2 ± 3.9	8.2 ± 2.9	7.5 ± 3.5	6.4 ± 6.1
Patellar shift (to lateral) [mm]	1.9 ± 2.9	2.4 ± 4.6	1.9 ± 1.7	2.4 ± 3.3	4.7 ± 5.0	5.2 ± 5.0
Patellar height [mm]	15.5 ± 8.0	15.3 ± 7.7	17.3 ± 8.4	16.7 ± 6.9	21.6 ± 7.9	21.0 ± 8.2
Femoro-patellar angle [°]	4.3 ± 4.1	7.4 ± 6.1	18.9 ± 6.4	17.4 ± 8.7	52.3 ± 4.9	51.4 ± 5.3
Tilt angle[°]	9.1 ± 5.3	9.5 ± 5.7	9.7 ± 4.6	9.5 ± 5.2	13.1 ± 9.1 *	14.0 ± 9.4 *
Patellar shift (to lateral) [mm]	2.1 ± 1.6	3.0 ± 2.1	2.4 ± 2.2	3.2 ± 3.1	6.7 ± 5.4 *	6.8 ± 5.3 *

* = Significant (*P*<0.05) difference compared to the healthy knees.

In PCL-insufficient knee joints both the patellar tilt and the patellar shift significantly increased (p < 0.05) at 90° flexion compared to the healthy control group, but not at 30° flexion and full extension (Table 1). In the sagittal plane, patellar height and patellofemoral angle did not differ from the results of healthy knee joints in all investigated positions. Flexing muscle activity led to a non-significant increase of the measured parameters in the transversal plane in PCL deficient knee joints.

## Discussion

The objective of the present study was to simultaneously investigate tibiofemoral and patellofemoral kinematics in patients with PCL-deficiency in comparison to healthy knee joints of the control group. At full extension and 30° flexion no significant difference was observed in PCL-deficient knee joints neither for the tibiofemoral nor for the patellofemoral kinematics. However, at 90° flexion the femur of PCL-deficient patients was positioned significantly more anteriorly in relation to the tibia. The patellar tilt angle as well as the patellar shift to the lateral side significantly increased compared to the healthy knee joints at 90° flexion, too. While no significant effect of isometric flexing muscle activity was observed in healthy individuals, in PCL-deficient knee joints an increased paradoxical anterior translation of the femur was observed at 90° flexion compared to the status of muscle relaxation. The results might underline the concept of the PCL being an important restraint to posterior tibial translation at flexion angles of more than 30° [3,9,23]. Since it`s known that increased tibiofemoral translation to the medial side may cause the development of medial OA these results might provide an explanation for the relatively high incidence of medial tibiofemoral and patellofemoral OA in patients with PCL-deficiency [4-7].

One limitation of the used technique is the fact, that weight bearing simulations are performed under static conditions. Since acquisition of a complete 3D MR data set requires a time period of approximately 4–5 minutes, dynamic studies can currently not be performed using the described 3D technique. “Real time” MRI, which means continuous MR imaging of moving objects in real time delivers actually only 2D images which implicates the problem of limited reproducibility and does not permit to measure displacement patterns three-dimensionally [24,25]. However, our results in healthy volunteers are consistent with findings in the literature of current in-vivo and in-vitro studies [26-31]. Therefore, we have the opinion that our results obtained under static loading conditions are not likely to interfere with the conclusions. One possibility to overcome this problem is the improvement of MRI sequences or the combination of MRI and orthogonal fluoroscopy allowing image acquisition under quasi-static weight-bearing flexion [13]. However, the latter technique is – admittedly to a small extent – suffering from radiation exposure to the patients.

Regarding tibiofemoral kinematics we found a posterior translation of the femur during knee flexion in healthy knee joints. The femoral roll back occurred predominantly on the lateral side, resulting in a medial pivoting motion. These findings are consistent with previously published kinematic analyses [26-31]. Furthermore, results in PCL-deficient knee joints show a more anterior position of the femur with respect to the tibia especially at higher degrees of flexion which is in keeping with previous in-vitro and in-vivo studies [2,32,33]. This paradoxical anterior translation of the femur at 90° flexion might be an expression of the clinically known fixed posterior position of the tibia in relation to the femur in patients with chronic PCL-deficiency [34]. In addition, our in vivo data confirm the findings of previous in vitro studies [33], that contraction of flexor muscle groups in PCL-deficient knees tends to result in an increased paradoxical anterior translation of the femur relative to the tibia at higher degrees of flexion.

Concerning patellar kinematics in healthy knees, Lee et al. [16] reported on a patellofemoral angle in the sagittal plane of about 22° at 30° flexion increasing to about 60° at 90° knee flexion, which is in accordance to our findings. Our results for patellar shifting and tilting are also consistent with previously published data [21,35,36]. However, many authors have reported on patellar kinematics in healthy knee joints, but only few in PCL-deficiency [10-12,14], although the influence of tibiofemoral kinematics on patellar motion in knee instability has been demonstrated [10,14-17]. Patellofemoral symptoms were described in about 50% of these patients [7,37]. Consistent to our findings van de Velde et al. [14] observed significant changes in patellofemoral kinematics at higher degrees of flexion especially in the transversal plane, too. However, in contrast to van de Velde et al. [14] who described a decrease of patellar shift (to the lateral side) we observed a significant increase of patellar shift and tilt angle at 90° flexion which tended to increase during isometric flexor muscle activity. Differences might occur due to different muscle loadings, thus applying 3 kg to the lower third of the shank in our experiment and supporting patient’s body weight on the leg being scanned in van de Velde’s experiment [14]. However, the increased patellar shift may be caused by the changes of the tibiofemoral joint with a decreased externally rotated and increased anteriorly positioned femur at 90° flexion which leads to a lateralization of the patella.

## Conclusion

In summary, in PCL-deficient knee joints significant differences could neither be observed for tibiofemoral nor for patellofemoral kinematics at full extension and 30° flexion. At 90° flexion tibiofemoral motion was significantly altered in terms of an increased anterior translation of the femur. During the external rotation from 30° to 90° flexion the amount of tibiofemoral translation increased medially and significantly decreased laterally leading to a reduced femoral external rotation during knee flexion. Additionally, the patellar tilt and shift to the lateral side were significantly increased compared to the healthy knee joints at 90° of flexion. During isometric muscle activity the amount of alterations (paradoxical femoral anterior translation, patellar tilt and shift) tended to increase compared to the status of muscle relaxation. We have the opinion, that these findings could support the concept of the PCL being an important restraint to posterior translation of the tibia at flexion angles of more than 30°. Future biomechanical studies must show, whether there is a direct influence between kinematic changes in PCL deficient knees and development of osteoarthritis.

## Competing interests

The authors declare that they have no competing interests.

## Authors’ contributions

RvER contributed to the conception and design of the study as well as the acquisition of data and was in charge of drafting and writing the manuscript. UL, SH and FP participated in the design of the study, performed the statistical analysis and helped to draft the manuscript. HG, MS and FW contributed to the conception and design of the study and gave intellectual feedback on the manuscript. RB contributed to the conception and the design of the study, the interpretation of data and gave intellectual feedback on the manuscript. All authors read and approved the final manuscript.

## Pre-publication history

The pre-publication history for this paper can be accessed here:

http://www.biomedcentral.com/1471-2474/13/231/prepub
